# Inhibition of HMGB1/RAGE-mediated endocytosis by HMGB1 antagonist box A, anti-HMGB1 antibodies, and cholinergic agonists suppresses inflammation

**DOI:** 10.1186/s10020-019-0081-6

**Published:** 2019-04-11

**Authors:** Huan Yang, Hui Liu, Qiong Zeng, Gavin H. Imperato, Meghan E. Addorisio, Jianhua Li, Mingzhu He, Kai Fan Cheng, Yousef Al-Abed, Helena E. Harris, Sangeeta S. Chavan, Ulf Andersson, Kevin J. Tracey

**Affiliations:** 1 Center for Biomedical Science The Feinstein Institute for Medical Research, 350 Community Drive, Manhasset, NY 11030 USA; 2 Center for Bioelectronic Medicine, The Feinstein Institute for Medical Research, 350 Community Drive, Manhasset, NY 11030 USA; 3 Elmezzi Graduate School of Molecular Medicine, Feinstein Institute for Medical Research, Northwell Health, Manhasset, NY USA; 4 Donald and Barbara Zucker School of Medicine at Hofstra/Northwell, Hempstead, New York, USA; 5 Center for Molecular Innovation, The Feinstein Institute for Medical Research, 350 Community Drive, Manhasset, NY 11030 USA; 6 Center for Molecular Medicine, Department of Medicine Solna, Karolinska Institute, 17176 Stockholm, Sweden; 7 Department of Women’s and Children’s Health, Karolinska Institute, Karolinska University Hospital, 17176 Stockholm, Sweden

**Keywords:** Cytokine, LPS, HMGB1, RAGE, Endocytosis sepsis

## Abstract

**Background:**

Extracellular high mobility group box 1 protein  (HMGB1) serves a central role in inflammation as a transporter protein, which binds other immune-activating molecules that are endocytosed via the receptor for advanced glycation end-products (RAGE). These pro-inflammatory complexes are targeted to the endolysosomal compartment, where HMGB1 permeabilizes the lysosomes. This enables HMGB1-partner molecules to avoid degradation, to leak into the cytosol, and to reach cognate immune-activating sensors. Lipopolysaccharide (LPS) requires this pathway to generate pyroptosis by accessing its key cytosolic receptors, murine caspase 11, or the human caspases 4 and 5. This lytic, pro-inflammatory cell death plays a fundamental pathogenic role in gram-negative sepsis. The aim of the study was to identify molecules inhibiting HMGB1 or HMGB1/LPS cellular internalization.

**Methods:**

Endocytosis was studied in cultured macrophages using Alexa Fluor-labeled HMGB1 or complexes of HMGB1 and Alexa Fluor-labeled LPS in the presence of an anti-HMGB1 monoclonal antibody (mAb), recombinant HMGB1 box A protein, acetylcholine, the nicotinic acetylcholine receptor subtype alpha 7 (α7 nAChR) agonist GTS-21, or a dynamin-specific inhibitor of endocytosis. Images were obtained by fluorescence microscopy and quantified by the ImageJ processing program (NIH). Data were analyzed using student’s t test or one-way ANOVA followed by the least significant difference or Tukey’s tests.

**Results:**

Anti-HMGB1 mAb, recombinant HMGB1 antagonist box A protein, acetylcholine, GTS-21, and the dynamin-specific inhibitor of endocytosis inhibited internalization of HMGB1 or HMGB1-LPS complexes in cultured macrophages. These agents prevented macrophage activation in response to HMGB1 and/or HMGB1-LPS complexes.

**Conclusion:**

These results demonstrate that therapies based on HMGB1 antagonists and the cholinergic anti-inflammatory pathway share a previously unrecognized molecular mechanism of substantial clinical relevance.

## Introduction

Sepsis is a life-threatening, systemic inflammation caused by dysregulated host responses to infection. It remains a major global cause of morbidity and mortality, and no targeted therapy is currently available (Singer et al. 2016). HMGB1, a ubiquitous nuclear protein present in all cell types, can be released into the extracellular milieu as a damage-associated molecular pattern molecule (DAMP) to act as a key endogenous and pathogenic mediator of sepsis. A large body of studies has demonstrated that HMGB1-specific antagonists are highly effective in experimental gram-negative sepsis, and most importantly that these agents can be successfully administered with exceptional delay (as late as 24 h after the initiation of sepsis) with maintained therapeutic effect (Yang et al. 2004; Qin et al. 2006). However, the molecular mechanisms underlying these beneficial effects by HMGB1 antagonists are not fully understood. Subsequent studies of experimental gram-negative sepsis have revealed that strategies based on anti-RAGE monoclonal antibodies or vagus nerve stimulation have similar delayed therapeutic action to HMGB1-specific antagonists (Liliensiek et al. 2004; Christaki et al. 2011; Huston et al. 2006, 2007; Rosas-Ballina et al. 2008). We reasoned that there may be common mechanisms for these therapeutic agents.

Toll-like receptor (TLR) 4 and RAGE are the dominant receptors used by extracellular HMGB1 to mediate inflammation (reviewed by Andersson et al. 2018a). In addition, extracellular HMGB1 augments inflammatory responses by binding other pro-inflammatory mediators including lipopolysaccharide (LPS), IL-1α and β, nucleic acids, histones and nucleosomes (Andersson et al. 2018b). The mechanism for the HMGB1-mediated synergy with the partner molecules is currently unknown. HMGB1 was demonstrated to be a critical mediator of endotoxin lethality two decades ago (Wang et al. 1999), and to bind the endotoxin lipid A region via two LPS-binding HMG box domains, the box A and B regions of HMGB1 (Youn et al. 2008, 2011). Furthermore, HMGB1 antagonists protect against LPS-mediated lethality in mice (Yang et al. 2004; Wang et al. 1999). Two recent studies have also demonstrated novel findings that significantly guide our understanding of the key HMGB1/LPS-mediated molecular mechanisms operating in the pathogenesis of sepsis (Xu et al. 2014; Deng et al. 2018). It was first demonstrated that HMGB1, acting through RAGE and dynamin-dependent signaling, is required for HMGB1 endocytosis, which in turn induces macrophage pyroptosis which occurs in vitro and also in vivo during endotoxemia (Xu et al. 2014). Studies by Deng et al. then established that extracellular HMGB1-LPS complexes bind to cell surface-expressed RAGE and are endocytosed to the endolysosomal compartment (Deng et al. 2018a). There HMGB1 permeabilizes the lysosomal membrane, a capacity exerted under acidic conditions, which enables LPS to reach its key pathogenic cytosolic receptor, caspase-11, to mediate inflammasome activation and pyroptosis (Broz and Dixit 2016). These observations demonstrated that RAGE-mediated internalization is a pivotal event in gram-negative sepsis, and prompted us to study approaches capable of blocking the cellular import of HMGB1/LPS and subsequent immune activation. We developed in vitro assays to screen agents that inhibited RAGE-dependent endocytosis in macrophages of HMGB1 or complexes of HMGB1 and LPS, and observed that anti-HMGB1 mAb, box A, acetylcholine and GTS-21 significantly inhibited RAGE-mediated HMGB1 or HMGB1-LPS complexes uptake.

## Materials and methods

### Materials

Recombinant mouse TNF, RAGE-Fc chimera protein and enzyme-linked immunosorbent assay (ELISA) kits were obtained from R & D System Inc. (Minneapolis, MN). Triton X-114, Lipopolysaccharide (LPS, *E. coli.* 0111:B4), peptidoglycan, pyridostigmine bromide, human macrophage-colony stimulating factor (M-CSF), acetylcholine chloride, GTS-21, 3, 3′, 5, 5′-Tetramethylbenzidine (TMB) substrate solution, lactate dyhydrogenase (LDH) cytotoxicity assay kit, non-immune rabbit IgG (Cat# I5006) and mouse IgG (Cat# I5381) were purchased from Sigma-Aldrich (St. Louis, MO). Ultrapure *E. coli* LPS (Cat # tlrl-pelps), poly I:C, and type B CpG oligonucleotide were obtained from InvivoGen (San Diego, CA). Thioglycollate medium was purchased from Becton Dickinson Co., (Sparks, MD). Fluorescent labeling kits were purchased from Molecular Probes (Eugene, OR). Alexa 568 labeled LPS was obtained from Invitrogen (Waltham, MA). Dynasore was purchased from Tocris Bioscience (Bristol, UK). Microscope cover glasses were obtained from Fisher Scientific (Cat# 12–545-82, Waltham, MA). Dako Fluorescence mounting medium was purchased from Agilent (Santa Clara, CA).

### Cell culture

Murine macrophage-like RAW 264.7 cells were obtained from American Type Culture Collection, (ATCC, Rockville, MD). Thioglycollate-elicited peritoneal macrophages were isolated from BALB/c mice (male, 8–12 weeks old) using a previously described method (Li et al. 2007). Cells were plated in 24-well plate, and treatment was carried out in serum-free Opti-MEM I medium (Life Technologies, Waltham, MA).

Human primary monocytes were purified by density gradient centrifugation through Ficoll from blood donated to the Long Island Blood Bank by healthy individuals (New York Blood Center, Melville, NY) (Yu et al. 2006). Cells were allowed to differentiate into macrophages for 7 days in complete DMEM medium containing M-CSF (1 ng/ml) in 24-well culture plate with microscope cover glasses. For cytokine measurements, macrophages were plated in 96-well plates and treatment was carried out in serum-free Opti-MEM I medium. Cells were incubated with stimuli (HMGB1, alone or with LPS; TLR3 agonist Poly I:C, TLR2 agonist PGN, and TLR9 agonist CpG DNA), plus increasing amounts of m2G7, box A, acetylcholine or GTS-21 (or IgG control) as indicated in the text for 16 h. Cell culture supernatants were collected for cytokine measurements.

### Expression and isolation of recombinant HMGB1 and box A, generation of HMGB1 redox isoforms, anti-HMGB1 antibodies and HMGB1/TLR4 antagonist K883

Recombinant HMGB1 (disulfide isoform) and box A were expressed in *E. coli* and purified to homogeneity as previously described (Yang et al. 2004; Li et al. 2004; Antoine et al. 2014). The integrity of HMGB1 proteins was verified by SDS-PAGE with Coomassie Blue staining, with the purity consistently over 85%. HMGB1 isoforms (disulfide, fully reduced and sulfonyl) were generated as previously described (Yang et al. 2012). LPS content was typically less than 1 pg/μg recombinant protein or un-detectable as measured by the Limulus assay. Polyclonal antibodies against HMGB1 B box were raised in rabbits, the titer was determined by immuno-blotting and the antibodies were affinity purified using cyanogen bromide activated Sepharose beads following standard procedure (Cocalico Biological, Inc. Reamstown, PA). Neutralizing activity of anti-HMGB1 was confirmed in macrophage cultures exposed to recombinant HMGB1 and assayed for ability to inhibit TNF release (Yang et al. 2004). Generation of neutralizing monoclonal antibodies to HMGB1 (m2G7) and epitope mapping were reported previously (Qin et al. 2006; Lundback et al. 2016). Clone m2G7 was found to bind a region between amino acids 53–63 in the box A region of HMGB1. Non-immune mouse IgG was used as isotype control in experiments where anti-HMGB1 antibody was used. HMGB1 antagonist K883 is a peptidomimetic analog of the tetra peptide P5779 (Yang et al. 2015a). K883 peptides were generated by Dr. Yousef Al-Abed (Feinstein Institute). The peptides were purified to 90% purity as determined by HPLC. Endotoxin was not detectable in the synthetic peptide preparations as measured by the Limulus assay. Peptides were first dissolved in DMSO and further diluted in PBS, and prepared freshly before each use.

### LPS measurements

Limulus assay, measuring LPS content, was performed following manufacturer’s instructions (BioWhittaker, Inc., Walkersville, MD). Contaminating LPS from protein preparations was removed by Triton X-114 extraction (Li et al. 2004).

### Quantitative PCR for mRNA measurements

For mRNA measurement, human primary macrophages were plated in 60 mm plates and treated in Opti-MEM I medium containing LPS +/− monoclonal anti-HMGB1 antibodies for 60 min. Cells were scraped off the plate and total RNA was isolated by Trizol method, in accordance with the manufacturer’s instructions (Tel-Test “B”, Inc., Friendswood, TX). Total RNA (1–4 μg) was reverse-transcribed to cDNA as previously described (Chaung et al. 2008). Primer pairs for human TNF and GAPDH were obtained from R&D systems (Cat # RDP-10 and RDP-39). Real time PCR was performed using 7300 Real-Time PCR system (Applied Biosystems, Foster City, CA) with SYBR green as detection dye. The gene expression is presented as fold change from the GAPDH level.

### Fluorescent microscopy for measurements of macrophage endocytic uptake

The labeling of HMGB1 with Alexa Fluor was performed according to the manufacturer’s instructions (Molecular Probes). To examine the uptake process of HMGB1, LPS, or complex of HMGB1 and LPS, RAW 264.7 cells, primary mouse or human macrophages seeded on glass coverslips were incubated with Alexa Fluor-labeled HMGB1, LPS or LPS plus HMGB1 in the presence or absence of increasing amounts of m2G7, box A, acetylcholine and GTS-21 simultaneously for 2 h at 37 °C. Acetylcholine esterase inhibitor pyridostigmine bromide was added (1 μM final concentration) whenever acetylcholine was used. As a positive control of endocytosis, cells were pre-incubated for 30 min with endocytosis inhibitor Dynasore (8 μM) (Saenz et al. 2014) before the addition of Alexa Fluor-labeled HMGB1 or LPS in some experiments. After incubation, cells were rinsed with phosphate buffered saline and fixed by using 4% PFA (paraformaldehyde) for 30 min at room temperature. Cells were mounted using permanent mounting medium containing 4′,6- diamidino-2-phenylinodole (DAPI) (Vecta mount from Vector laboratories, Burlingame, CA). Images were taken by Carl Zeiss fluorescence microscope with a 40 x objective and quantified using the Image J program (NIH, Gov).

### Cytokine measurements

Levels of TNF and IL-6 released in the cell culture supernatants or mice sera were measured by commercially obtained ELISA kits (Cat # DY410 for mouse TNF, DY401 for mouse IL-1β and DY406 for mouse IL-6, DY210 for human TNF and DY206 for human IL-6) according to the instructions of the manufacturer (R & D System).

### ELISA demonstrating box A binding to RAGE

Human RAGE–Fc chimera protein (0.25 nM) was incubated with or without box A (10 μM) in PBS containing 0.05% Tween 20 at room temperature for 30 min. The mixture was then added to HMGB1 coated plates (60 nM solution on Maxisorp plate from Nunc, (Thermos Fisher)) and incubated for an additional 100 min. After washing the plate with PBS containing 0.05% Tween 20, anti-human HRP antibody diluted in 1% BSA in PBS was added (1:2000 dilution, Dako, Agilent Technologies). After incubation at room temperature for 90 min, the plate was washed and TMB substrate solution was added. The reaction was stopped by adding 2 N H_2_SO_4_ and the plate was subjected to reading at 450 nm.

### Animals

BALB/c mice (male 8–12 weeks old) were purchased from Taconic Laboratories (Hudson, NY). Mice were allowed to acclimate for 7 days before experiments. All animal procedures were approved by the Feinstein Institute for Medical Research Institutional Animal Care and Use Committee (IACUC). Mice were housed in the Center for Comparative Physiology of the Feinstein Institute for Medical Research under standard temperature, light and dark cycle conditions.

### LPS toxicity

BALB/c mice (male, *n* = 5–10 in each group) were given intraperitoneal (IP) injection of 500 μg/mouse polyclonal rabbit anti-HMGB1 antibody at various time points, and were challenged with 7.5 mg/kg LPS injected IP. Two hours later, mice were euthanized, serum was collected, and TNF was measured by ELISA. In some experiments, mice were given an LD_75_ dose of LPS (7.5 mg/kg) injected IP and treated with anti-HMGB1 monoclonal antibodies m2G7 (50 μg/mouse) administered IP either simultaneously or prior to LPS injection at the times indicated in the text.

### Statistical analysis

Data are presented as means + SEM. Differences between treatment groups were determined by Student’s t test. One-way ANOVA was used for multiple comparisons. For post-hoc analysis, we used the least significance test for groups less than 4 and the Tukey test for groups more than 4. *P* < 0.05 was considered statistically significant.

## Results

### Polyclonal as well as monoclonal anti-HMGB1 antibodies reduced LPS-induced TNF release in vivo and in vitro

**Fig. 1:**
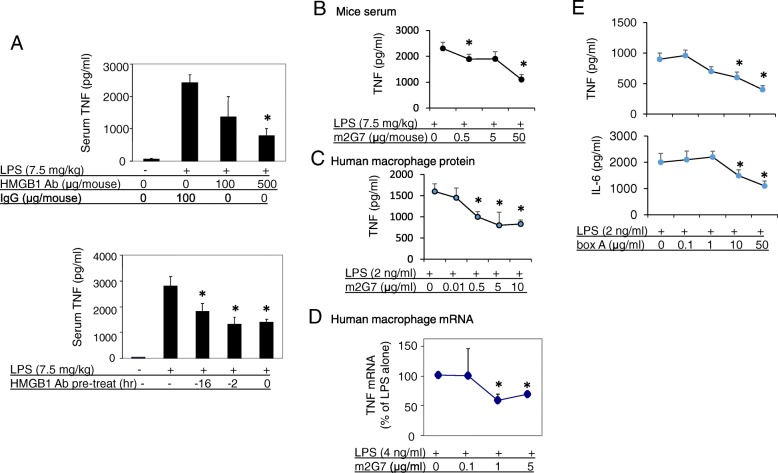
Anti-HMGB1 antibodies attenuated LPS-induced TNF release in vitro and in vivo*.* **a**. Upper: Male BALB/c mice received IP injection of polyclonal rabbit anti-HMGB1 antibody or rabbit control IgG as indicated at time point − 16 h, LPS (7.5 mg/kg) at 0 h (IP injection). Mice were euthanized 2 h post LPS administration, and serum TNF was assayed. *N* = 6–8 mice per group. *: *P* < 0.05 vs. IgG group. Lower: Male BALB/c mice received IP injection (500 μg/mouse) of polyclonal rabbit anti-HMGB1 antibody at indicated time points, and were challenged with LPS (7.5 mg/kg, IP injection). Mice were euthanized 2 h post LPS injection, serum TNF levels were measured by ELISA. *N* = 5–10 per group. *: *P* < 0.05 vs. LPS alone. **b**. Male BALB/c mice had IP injection of m2G7 and LPS and were euthanized 2 h later. Serum TNF was measured. *N* = 10 mice per group. *: *P* < 0.05 vs. LPS alone. **c**. Primary human macrophages were seeded at a density of 10^5^ cells/well in 24-well culture plates and were stimulated with LPS (2 ng/ml) in the presence or absence of m2G7 for 16 h in serum-free Opti-MEM medium. TNF released in the supernatant was measured. *N* = 3–4 separate experiments each from different donors. *: *P* < 0.05 vs. LPS alone. **d**. Primary human macrophages were plated in 60 mm plates (10^6^ cells/well) and stimulated with LPS in the presence or absence of m2G7 for 60 min. Total RNA was isolated, TNF gene expression was examined and expressed as fold change from the GAPDH level. *N* = 4–5 experiments each from different donors. Data are expressed as percent of LPS stimulation alone. *: *P* < 0.05 vs. LPS alone. **e**. Primary human macrophages in 96-well plate (5 × 10^4^ cells/well) were activated with LPS in the presence or absence of box A for 2 h at 37 °C. TNF (upper) and IL-6 (lower) released were measured. N = 4 separate experiments each from different donors. *: *P* < 0.05 vs. LPS alone

While studying the mechanism of HMGB1, we observed that administration of neutralizing anti-HMGB1 antibodies ameliorated LPS-mediated toxicity in mice (Fig. 1a, b). Treatment with polyclonal anti-HMGB1 antibodies significantly reduced LPS-induced TNF release dose-dependently as compared to IgG-treated control mice. Time course experiments showed that the inhibition effects on LPS-induced TNF release by polyclonal anti-HMGB1 antibodies were similar among multiple time points of administration (Fig. 1a). To test the specificity of these effects, we repeated the experiments with a monoclonal neutralizing anti-HMGB1 antibody (m2G7) (Qin et al. 2006; Lundback et al. 2016) which generated highly analogous results (Fig. 1b). In parallel, in vitro studies showed that TNF release in primary human macrophage cultures activated by LPS was dose-dependently inhibited by the addition of m2G7 (Fig. 1c). The HMGB1 antagonist inhibited both TNF mRNA (Fig. 1d) and TNF protein expression without affecting cell viability, as assessed by the LDH release assay (data not shown). Thus, administration of anti-HMGB1 antibodies inhibited LPS-induced TNF levels in vitro and during endotoxemia.

### Recombinant HMGB1 box A protein reduced LPS-stimulated TNF and IL-6 release in primary macrophage cultures

Experiments with primary human macrophages demonstrated that another HMGB1-specific antagonist, recombinant box A, suppressed LPS-induced TNF, as well as IL-6, release in a dose-dependent manner (Fig. 1e) (Yang et al. 2004). Thus, these results confirmed that different HMGB1-specific antagonists counteracted LPS-induced pro-inflammatory cytokine release.

**Fig. 2:**
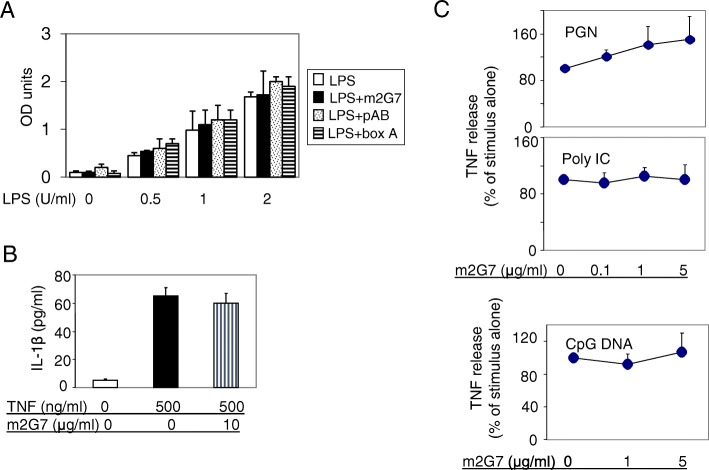
Neither anti-HMGB1 antibodies nor box A suppressed LPS activity in the Limulus assay; m2G7 did not suppress pro-inflammatory cytokine release induced by TNF, peptidoglycan (PEG), Poly I: C or CpG DNA from human macrophages. **a**. Polyclonal anti-HMGB1 antibody (pAB, 1 µg/ml), m2G7 (1 μg/ml) or box A (10 μg/ml) were added to LPS (from 0 to 2 U/ml) solution and incubated at 37 °C for 30 min and limulus assay was performed. Results are representative of 2 separate measurements each done in triplicate. **b**. Primary human macrophages in 96-well culture plate (5 × 10^4^ cells/well) were stimulated with mouse TNF in the presence or absence of m2G7 for 16 h. IL-1β released was measured. *N* = 3 experiments. **c**. Primary human macrophages in 96-well culture plate (5 × 10^4^ cells/well) were stimulated with TLR 2 agonist PGN (5 μg/ml), TLR3 agonist Poly I:C (50 μg/ml) or TLR9 agonist CpG DNA (1 μM) for 16 h. TNF released was measured. N = 3 separate experiments

To clarify whether any of the studied HMGB1-specific antagonists bound directly to LPS we performed a Limulus assay to measure LPS activity in the presence or absence of HMGB1 antibodies or box A. None of the HMGB1 antagonists altered the LPS activity in the assay, contradicting direct LPS neutralization as the cause of the observed effects on downregulated LPS-induced cytokine release (Fig. 2a).

### Monoclonal anti-HMGB1 antibodies did not suppress pro-inflammatory cytokine release induced by TNF, peptidoglycan (PGN), poly I:C or CpG DNA in primary macrophage cultures

To further examine the specificity of m2G7 on LPS-induced cytokine release, cultured primary human macrophages were stimulated by TNF in the presence or absence of m2G7. Addition of m2G7 did not significantly modify TNF-induced IL-1β release (Fig. 2b). Furthermore, TNF release was not significantly altered by m2G7 in primary macrophage cultures stimulated by other TLR ligands, such as the TLR 2 agonist PGN, the TLR3 agonist Poly I:C, or the TLR9 agonist CpG DNA (Fig. 2c). Taken together, these results indicate that HMGB1 was selectively involved in the LPS signaling pathway in our experiments.

### Box A protein and the anti-HMGB1 mAb m2G7 each inhibited HMGB1 endocytosis in macrophages

**Fig. 3:**
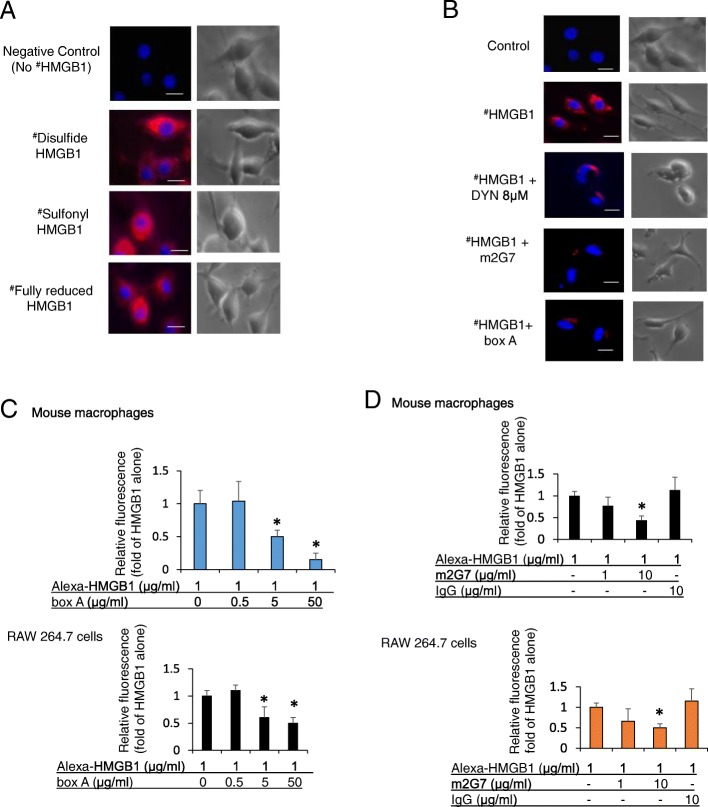
Anti-HMGB1 m2G7 and box A inhibited macrophages endocytosis of HMGB1. **a**. Murine macrophage-like RAW 264.7 cells seeded on 24-well culture plate on cover slips (1 × 10^4^/well) were incubated with Alexa 555 labeled (red) isoforms of HMGB1 (disulfide, sulfonyl and fully reduced) for 2 h at 37 °C. Endocytic uptake of HMGB1 was visualized via fluorescence microscope. Nuclei were counterstained with DAPI (blue). Right panel shows corresponding phase contrast image of cells. Data are representative from 4 independent experiments. Scale bar = 10 μm. **b**. RAW 264.7 cells seeded on 24-well culture plate on cover slips (1 × 10^4^/well) were incubated with Alexa 555 labeled HMGB1 (^#^ red) alone or plus Dynasore (DYN, 8 μM), m2G7 (5 μg/ml), or box A (50 μg/ml) for 2 h at 37 °C. Endocytic uptake of HMGB1 was visualized. Right panel shows corresponding phase contrast image of cells. Data are representative from 3 to 4 independent experiments. Scale bar = 10 μm. **c**. Thioglycollate-elicited mouse primary macrophages (upper) or RAW 264.7 cells (lower) were seeded on 24-well culture plate on cover slips (1 × 10^4^/well) and incubated with Alexa 555-HMGB1, alone or with box A for 2 h at 37 °C. HMGB1 endocytosis results expressed as fold of Alexa 555-HMGB1 alone. *N* = 30–42 cells per treatment. *: *P* < 0.05 vs. HMGB1 alone. **d**. Thioglycollate-elicited mouse primary macrophages (upper) or RAW 264.7 cells (lower) were seeded on 24-well culture plate on cover slips (1 × 10^4^/well) and incubated with Alexa 555 labeled HMGB1 (1 μg/ml) alone or with m2G7 or mouse IgG for 2 h at 37 °C. Endocytosis results expressed as fold of Alexa 555-HMGB1 alone. *N* = 32–44 cells per treatment. *: *P* < 0.05 vs. HMGB1 alone

Recognizing that RAGE-mediated endocytosis of LPS-HMGB1 complexes is key in the pathogenesis of gram-negative sepsis (Deng et al. 2018a), encouraged us to develop a cellular assay to examine RAGE-mediated HMGB1 endocytosis in macrophage cultures. This assay allowed us to study the intracellular uptake and to identify molecules capable of inhibiting endocytosis. Fluorochrome-labeled (Alexa 555) HMGB1 redox isoforms were incubated with cultured macrophages for 2 h at 37 °C and the intracellular uptake was recorded by microscopy in fixed cells. Our pilot experiments demonstrated that labeled disulfide, sulfonyl and fully reduced HMGB1, respectively, were all internalized (Fig. 3a). All subsequent studies were then conducted using Alexa 555-labeled disulfide HMGB1, since this is the most relevant pro-inflammatory isoform. Initial studies performed in murine macrophage-like RAW 264.7 cells demonstrated that box A, m2G7 and Dynasore, prevented endocytosis, as each significantly inhibited the intracellular uptake of labeled HMGB1 (Fig. 3b-d). In contrast, HMGB1 internalization was not reduced in cultures supplemented with K883 (at 50 μg/ml, data not shown), a specific inhibitor of HMGB1/TLR4-binding (Yang et al. 2015a).

The endocytosis of labeled HMGB1 was dose-dependently reduced by box A in primary mouse macrophages (Fig. 3c, upper). The relative fluorescence intensity for Alexa 555 HMGB1 at 1 μg/ml = 1.0 + 0.2, HMGB1 + box A 0.5 μg/ml =1.1 + 0.3, HMGB1 + box A 5 μg/ml = 0.5* + 0.1. HMGB1 + box A 50 μg/ml = 0.15* + 0.1. *: *P* < 0.05 vs. Alexa 555 HMGB1 alone. Similar blocking effects by box A were confirmed in murine macrophage-like RAW 264.7 cultures (Fig. 3c, lower). The m2G7 antibody (but not IgG) also dose-dependently reduced HMGB1 endocytosis in both RAW 264.7 cells (Fig. 3d upper) and primary mouse macrophages (Fig. 3d lower). The relative fluorescence intensity for Alexa 555 HMGB1 at 1 μg/ml = 1.0 + 0.1, HMGB1 + m2G7 1 μg/ml = 0.6 + 0.2, HMGB1 + m2G7 10 μg/ml = 0.4* + 0.08. *: *P* < 0.05 vs. Alexa 555-HMGB1 alone (Fig. 3c upper). When compared on a molar basis, 25X molar excess of box A and 2X of m2G7 resulted in 60–70% reduction of endocytosed HMGB1. Together, these results indicate that the m2G7 and box A both specifically block endocytic uptake of HMGB1 in macrophages.

### Recombinant box A protein binds to RAGE and prevents full length HMGB1 from binding to RAGE

**Fig. 4:**
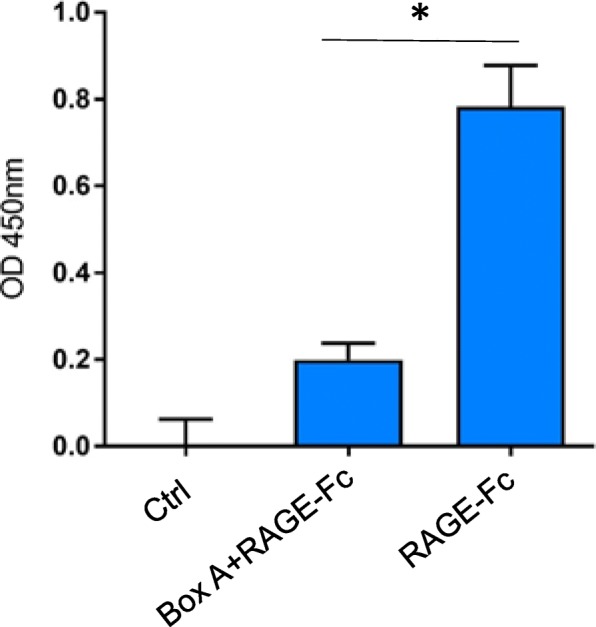
ELISA demonstrating box A binding to RAGE. Soluble RAGE-Fc chimera (0.25 nM) was incubated with an excess amount of box A (10 μM) at room temperature for 30 min. The mixture was then added to HMGB1 coated plate (60 nM) and incubated for 100 min at room temperature. After washing the plates, anti-human HRP antibody was added and incubated for 90 min at room temperature. Optical density (OD) at 450 nm was measured. Ctrl = No addition of RAGE-Fc. *: *P* < 0.0001

These results raised the question about what is the mode of action for box A- or m2G7 -mediated inhibition of HMGB1 internalization. Previous studies have established that RAGE is required for HMGB1 endocytosis (Xu et al. 2014; Deng et al. 2018a) and that HMGB1 has two RAGE-binding regions, one in the box A (sequence 23–50) (LeBlanc et al. 2014) and one in the box B domain (sequence 150–183) (Huttunen et al. 2002). We hypothesized that recombinant box A protein might compete with full length HMGB1 for RAGE binding, leading to the inhibited endocytosis. We constructed a binding assay with plates coated with full length HMGB1 and added a recombinant human RAGE-Fc chimeric protein that was detected by an anti-human IgG Fc-specific labeled antibody. A pre-incubation of the chimeric RAGE protein with box A greatly reduced its capacity for subsequent HMGB1 binding (Fig. 4). These results indicated that the box A domain is a functionally important RAGE-binding domain. It is thus noteworthy that the binding site of the neutralizing anti-HMGB1 m2G7 is HMGB1 sequence 53–63 (Qin et al. 2006), which is in close proximity to the identified RAGE-binding site in the box A domain. Thus box A and the anti-HMGB1 mAb both block HMGB1-RAGE binding.

### Acetylcholine and the selective α-7 nicotinic acetylcholine receptor agonist GTS-21 each inhibited HMGB1 endocytosis in macrophages

**Fig. 5:**
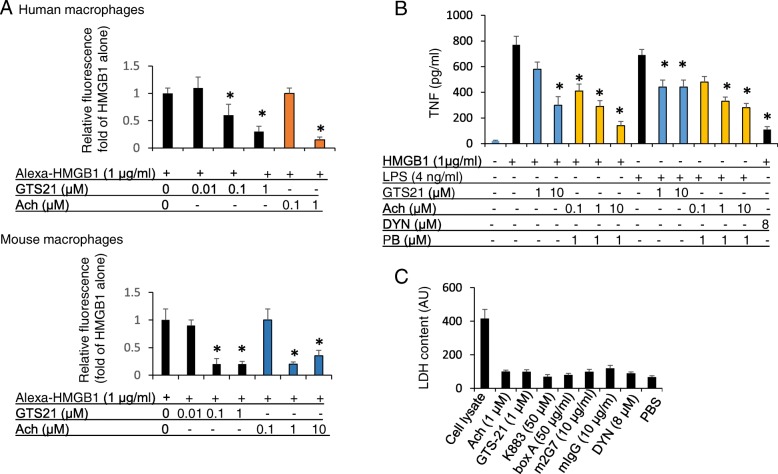
Cholinergic agonists acetylcholine and GTS-21 inhibited HMGB1 endocytosis, reduced HMGB1 and LPS-induced TNF release in macrophages. **a**. Primary human macrophages (upper) or thioglycollate-elicited mouse macrophages (lower) seeded on 24-well culture plate on cover slips (1 × 10^4^/well) were incubated with Alexa 555- HMGB1 (1 μg/ml) alone or plus GTS-21 or acetylcholine for 2 h at 37 °C. Endocytosis results of Alexa 555-HMGB1 were expressed as fold of Alexa 555-HMGB1 alone. *N* = 32–48 cells per treatment. *: *P* < 0.05 vs. Alexa 555-HMGB1 alone. **b**. RAW 264.7 cells in 96-well culture plates were used at 90% confluence. Cells were stimulated with HMGB1 or LPS, with the addition of GTS-21, acetylcholine (Ach) as indicated for 16 h. Pyridostigmine bromide (PB) was added (1 μM). TNF released was measured using ELISA. N = 3 experiments. *: *P* < 0.05 vs. HMGB1 or LPS alone. **c**. RAW 264.7 cells in 96-well culture plates (90% confluence) were incubated with Ach, GTS-21, K883, box A, m2G7, mouse IgG (mIgG), or Dynasore (DYN) as indicated for 16 h. Cell lysate (positive control) or supernatants were collected and assayed for LDH content (AU). AU: arbitrary unit. N = 3

We next decided to study cholinergic agents in our HMGB1 endocytosis assay because both electrical vagus nerve stimulation (Huston et al. 2007) and systemic administration of GTS-21 (Rosas-Ballina et al. 2008) treat gram-negative sepsis even after significantly delayed administration, like the HMGB1 antagonists. We used primary human macrophages and found that both acetylcholine and GTS-21 dose-dependently inhibited endocytosis of the labeled HMGB1 (Fig. 5a, upper). Experiments with GTS-21 generated the following results: the relative fluorescence intensity for Alexa 555 HMGB1 at 1 μg/ml = 1.0 + 0.1, HMGB1 + GTS-21 0.1 μM = 0.6 + 0.2*, HMGB1 + GTS-21 1 μM = 0.3* + 0.1. *: *P* < 0.05 vs. HMGB1 alone. For acetylcholine experiments: HMGB1 + acetylcholine 0.1 μM = 1 + 0.1, HMGB1 + acetylcholine 1 μM = 0.15* + 0.06. *: *P* < 0.05 vs. Alexa-HMGB1 alone. These findings were confirmed in primary mouse macrophage cultures (Fig. 5a lower), and also further verified in RAW 264.7 cells (Data not shown). Acetylcholine and GTS-21 not only inhibited HMGB1 endocytosis, but also suppressed TNF release after HMGB1 or LPS stimulation (Fig. 5b). Inhibiting endocytosis by Dynasore also significantly reduced HMGB1-induced TNF release, demonstrating that this is an endocytosis-related event. In addition, LPS-induced TNF was also dose-dependently reduced by GTS-21 and acetylcholine (Fig. 5b). Cell viability was not significantly affected by these HMGB1 antagonists or cholinergic agents as assessed by the LDH release assay (Fig. 5c).

### HMGB1 enhanced endocytosis of LPS, while anti-HMGB1 m2G7, box A protein and acetylcholine inhibited endocytosis of the LPS-HMGB1 complexes

**Fig. 6:**
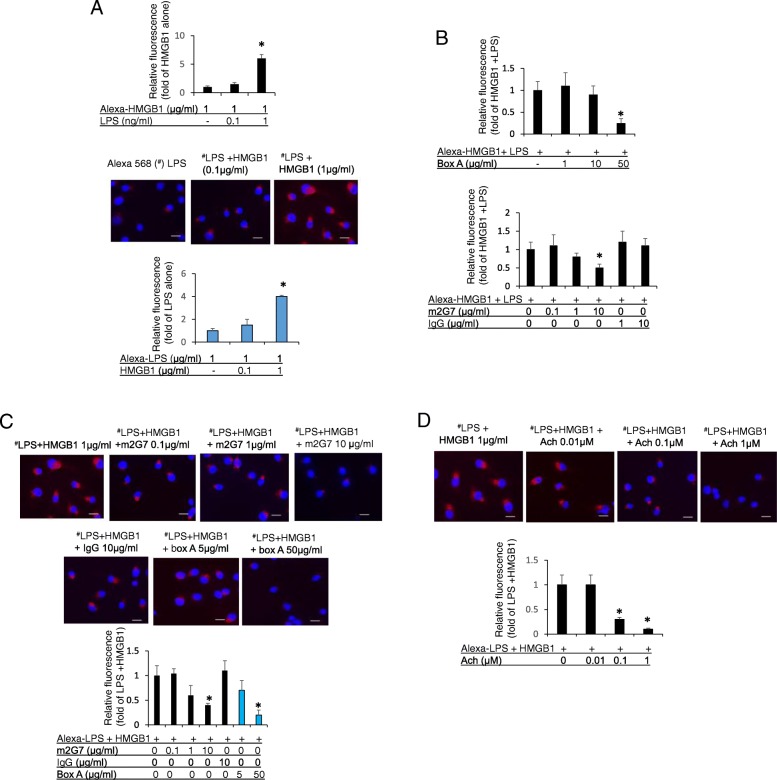
Macrophage endocytosis of LPS-HMGB1 complexes was inhibited by m2G7, box A and acetylcholine. **a**. Upper: RAW 264.7 cells, seeded on 24-well culture plate on cover slips (1 × 10^4^/well), were incubated with Alexa 555 labeled HMGB1 (1 μg/ml) alone or in combination with increasing amounts of LPS as indicated for 2 h at 37 °C. Endocytic results were expressed as fold change of Alexa 555-HMGB1 alone. *N* = 30–35 cells per treatment and are representative from 6 independent experiments. Middle and lower: RAW 264.7 cells, seeded on 24-well culture plate on cover slips (1 × 10^4^/well), were incubated with Alexa 568 labeled LPS (^#^ red, 1 μg/ml) in complex with HMGB1 as indicated. *N* = 28–30 cells for each treatment. Endocytic uptake was expressed as fold change of Alexa 555-HMGB1 (upper) or Alexa 568-LPS (lower) alone. Scale bar = 10 μm. *: *P* < 0.05 vs. Alexa 555-HMGB1 or Alexa 568-LPS alone. **b**. RAW 264.7 cells, on 24-well culture plate on cover slips (1 × 10^4^/well), were incubated with Alexa 555-HMGB1 (1 μg/ml) in complex with LPS (1 ng/ml), plus m2G7, mouse IgG or box A as indicated for 2 h at 37 °C. Endocytic uptake of Alexa 555-HMGB1 was expressed as fold change of Alexa 555-HMGB1 + LPS. *: *P* < 0.05 vs. Alexa 555-HMGB1 + LPS. N = 30–40 cells for each treatment. **c**. RAW 264.7 cells, on 24-well culture plate with cover slips (1 × 10^4^/well), were incubated with Alexa 568 labeled LPS (^#^ red, 1 μg/ml) in complex with HMGB1 (1 μg/ml), together with m2G7, mouse IgG or box A as indicated for 2 h at 37 °C. Endocytic uptake of Alexa 568 LPS was expressed as fold of Alexa 568-LPS + HMGB1. Scale bar = 10 μm. *: *P* < 0.05 vs. Alexa 568-LPS + HMGB1. N = 30–35 cells for each treatment. **d**. RAW 264.7 cells, seeded on 24-well culture plate on cover slips (1 × 10^4^/well), were incubated with Alexa 568 labeled LPS (^#^ red, 1 μg/ml) in complex with HMGB1 (1 μg/ml), together with acetylcholine as indicated for 2 h at 37 °C. Endocytic uptake of Alexa 568 LPS was visualized, analyzed and quantified using Image J. *: *P* < 0.05 vs. Alexa 568-LPS + HMGB1. N = 30–35 cells for each treatment. Scale bar = 10 μm

Endocytosis in RAW 264.7 cells of fluoro-labeled (Alexa 568) LPS was enhanced in the presence of extracellular HMGB1 (Fig. 6a upper). These results support our previous findings that HMGB1 and LPS act in synergy to enhance endocytosis of each other (Fig. 6a middle and lower). In cultured RAW 264.7 cells, both box A and anti-HMGB1 m2G7 dose-dependently inhibited endocytosis of the HMGB1 and fluoro-labeled LPS complexes, whereas control IgG did not (Fig. 6b). HMGB1 reciprocally and dose-dependently enhanced Alexa 568-labeled LPS endocytosis and box A protein, as well as m2G7, inhibited the endocytosis of these LPS-labeled HMGB1 complexes (Fig. 6a, c). Likewise, the addition of acetylcholine led to significantly reduced uptake of HMGB1/LPS complexes (Fig. 6d). Thus, anti-HMGB1 m2G7, box A protein and acetylcholine share a capacity to block intracellular uptake of extracellular HMGB1/LPS complexes.

### Anti-HMGB1 m2G7 and box A protein each inhibited HMGB1/LPS complexes-induced TNF release from macrophages

**Fig. 7:**
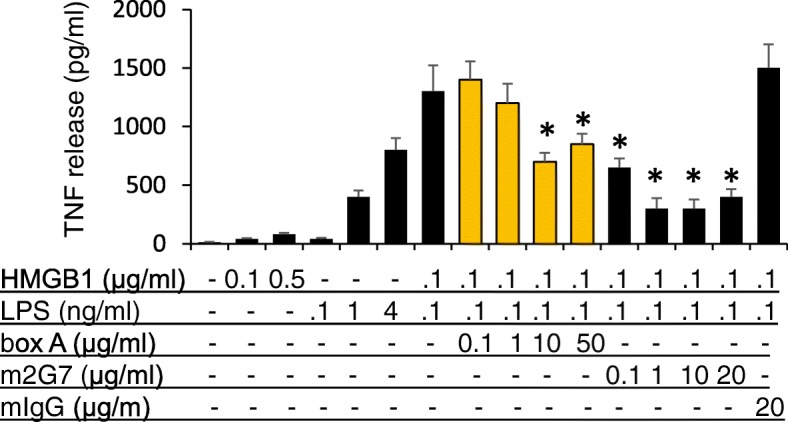
TNF release induced by LPS-HMGB1 complexes in RAW 264.7 cells was reduced by box A and m2G7. RAW 264.7 cells in 96-well culture plates were used at 90% confluence. Cells were stimulated with HMGB1 or LPS alone or HMGB1+ LPS, with the addition of box A, m2G7 or mouse IgG as indicated for 16 h. TNF released was measured. N = 3 experiments. *: *P* < 0.05 vs. HMGB1 + LPS

To study the functional consequences of modified endocytosis, we next examined whether inhibiting LPS/HMGB1 endocytosis also blocked the subsequent inflammatory cytokine release. By adding a complex generated by inactive quantities of HMGB1 (0.1 μg/ml) and LPS (0.1 ng/ml) to macrophage-like RAW 264.7 cells, we observed synergistically enhanced TNF release in the supernatants (TNF release without stimulation = 10 + 6 pg/ml, HMGB1 at 0.1 μg/ml = 40 + 7 pg/ml, LPS at 0.1 ng/ml = 41 + 8, HMGB1 0.1 μg/ml + LPS 0.1 ng/ml = 1300* + 222 pg/ml. *: *P* < 0.05 vs. HMGB1 or LPS alone) (Fig. 7). Both Box A and m2G7 mediated dose-dependent and significant inhibition of TNF release stimulated by the HMGB1/LPS complexes, whereas control IgG did not.

**Fig. 8:**
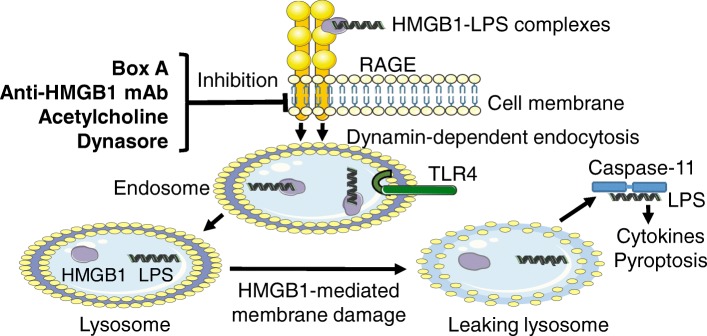
Molecules inhibiting RAGE-mediated endocytosis of LPS-HMGB1 complexes. The pathogenesis of endotoxemia is caused by extracellular LPS getting attaching to extracellular HMGB1 and forming a complex that is endocytosed via RAGE-dependent pathway. Both LPS and HMGB1 then activate endosomal TLR4 receptors before being transferred to the lysosomal system. HMGB1 permeabilizes lysosomal membranes under acidic conditions and thus allows LPS to avoid lysosomal degradation and leaks out into the cytosol (Deng et al. 2018a). LPS is thus enabled to activate its key cytosolic receptor, caspase-11 in murine macrophages to induce pyroptosis. The discoveries of the present study are that box A, anti-HMGB1 m2G7 and acetylcholine each inhibits the cellular internalization of LPS-HMGB1 complexes and subsequent immune activation

These results demonstrate that box A, m2G7, acetylcholine, and dynasore all inhibited endocytosis of extracellular HMGB1 or LPS/HMGB1 complexes and subsequent immune activation (Figs. 7 and 8).

## Discussion

The recent findings by Deng et al showed that LPS depends on HMGB1- and RAGE-assisted cellular transport. HMGB1 opens up lysosomes and allows LPS leakage into cytosol to reach and activate its key receptor caspase-11. This drives the pathogenesis of endotoxemia and also incites a clinical interest in how this data can be applied to improve outcomes in gram-negative sepsis (Deng et al. 2018a). This group uncovered the critical importance of the LPS-HMGB1-RAGE cooperation and also confirmed previous studies that m2G7 prevents mortality in experimental gram-negative sepsis, but they did not elucidate its mode of action (Qin et al. 2006; Deng et al. 2018a). Identifying agents capable of inhibiting RAGE-mediated endocytosis is the scope of the present study. Our main discoveries are that the m2G7, recombinant box A, acetylcholine and GTS-21 all prevent internalization of HMGB1, as well as HMGB1/LPS complexes (m2G7, recombinant box A, acetylcholine only), in cultured macrophages (Fig. 8). In comparison, a specific HMGB1 inhibitor (K883) targeting HMGB1/TLR4-mediated pathway did not inhibit this internalization (Yang et al. 2015a). It is known that all the studied agents are highly protective in experimental gram-negative sepsis, even after delayed administration, leaving several questions about the therapeutic mechanisms unanswered (Yang et al. 2004; Qin et al. 2006; Huston et al. 2007; Rosas-Ballina et al. 2008; Deng et al. 2018a; Yang et al. 2015a). Blocking RAGE-mediated endocytosis of HMGB1 and LPS complexes down-regulates immune activation and pyroptosis, which are key elements causing lethality in gram-negative sepsis (Deng et al. 2018a).

Redox conversions of HMGB1 represent important functional post-translational modifications determining receptor usage for extracellular HMGB1 (Andersson et al. 2018b; Yang et al. 2015b). However, it is presently unknown whether RAGE-HMGB1 interactions are influenced by the redox state of HMGB1. Our pilot experiments demonstrated that fully reduced, disulfide and sulfonyl HMGB1 were similarly well taken up by cultured macrophages. All subsequent endocytosis studies were performed with fluoro-labeled disulfide HMGB1, as it has been shown to be the most potent form of HMGB1 in inflammatory disorders (Yang et al. 2015a).

Recombinant box A is a successful therapeutic HMGB1 antagonist in many experimental models of both sterile and infectious tissue inflammation (Yang et al. 2004; Andersson et al. 2018a; Yang et al. 2015b; Andersson and Tracey 2011). However, its mode of action has never been elucidated. This knowledge gap and the lack of a bioassay to verify the bioactivity of a given batch of box A have so far precluded further clinical development. Our discovery that box A binds to RAGE and competes with full length HMGB1 binding to RAGE provides an attractive explanation for its mode of action in the prevention of RAGE-mediated endocytosis of HMGB1-partner molecule complexes and subsequent immune activation. The endocytosis assay and ELISA binding assay used by our group could now be established for further clinical development of box A.

HMGB1 amino acid sequence 23–50 in the box A domain is a critical region for HMGB1 binding to RAGE enabling endocytosis, which agrees with our findings that m2G7 acts as a potent inhibitor of HMGB1 internalization via RAGE as well (LeBlanc et al. 2014). The m2G7 binds to sequence 53–63 of the box A domain in close proximity to the RAGE-binding region, and it is thus likely that steric hindrance by the bound antibody may preclude HMGB1 from RAGE attachment (Qin et al. 2006). We also demonstrated that recombinant box A bound to RAGE and prevented full-length HMGB1 from binding to RAGE (Fig. 4). We propose that both box A and the m2G7 block HMGB1-RAGE binding as the therapeutic mode of action.

The cholinergic anti-inflammatory pathway plays an important functional role in the prevention of exaggerated inflammation, including that which occurs in gram-negative sepsis (Pavlov et al. 2018). The effector molecule in this neural circuit is acetylcholine acting via the nicotinic acetylcholine receptor subunit 7 to suppress the activation of the regulatory transcription factor nuclear factor-kB (NF-kB) which activates innate immune responses. We have not yet identified the molecular mechanisms mediating the acetylcholine- and GTS-21-inhibited RAGE-dependent endocytosis of HMGB1 and HMGB1-LPS complexes (Ach only). RAGE is a pro-inflammatory type I transmembrane receptor and a member of the immunoglobulin gene superfamily. RAGE is present in many cell types mainly in a preformed intracellular pool that can be rapidly transported to the cell surface during cell activation (Ramasamy et al. 2011) and this translocation is partly under NF-kB control (Bierhaus et al. 2005; Kierdorf and Fritz 2013). Together, these findings indicate a central role for RAGE in transporting HMGB1 and LPS complexes to activate cytosolic receptors during inflammation independent of TLR4 and that this is inhibited by anti-HMGB1 agents and acetylcholine.

## Conclusion

We have demonstrated that an anti-HMGB1 mAb, HMGB1 antagonist box A protein, acetylcholine, and the nicotinic acetylcholine receptor subtype alpha 7 agonist GTS-21 all restricted the cellular internalization of HMGB1 and HMGB1-LPS complexes (anti-HMGB1 mAb, HMGB1 antagonist box A protein, acetylcholine only) in cultured macrophages. Our findings reveal a shared mechanism for these experimental therapeutic pathways and provide a previously unrecognized rationale for the treatment of multiple inflammatory diseases of either infectious or sterile origin.

## Abbreviations

DAMP: Damage-associated molecular pattern molecule
DAPI: 4′6-diamidino-2-henylinodole
ELISA: Enzyme-linked immunosorbent assay
HMGB1: High mobility group box 1 protein
IL-6: Interleukin-6
IP: Intraperitoneal
LPS: Lipopolysaccharide
M-CSF: Macrophage-colony stimulating factor
NF-κB: Nuclear factor - κB
PFA: Paraformaldehyde
PGN: Peptidoglycan
RAGE: Receptor for advanced glycation end-products
TLR4: Toll-like receptor 4
TNF: Tumor necrosis factor
α7 nAchR: α7 nicotinic acetylcholine receptor
